# Efficacy of quetiapine for delirium prevention in hospitalized older medical patients: a randomized double-blind controlled trial

**DOI:** 10.1186/s12877-021-02160-7

**Published:** 2021-03-31

**Authors:** Saran Thanapluetiwong, Sirasa Ruangritchankul, Orapitchaya Sriwannopas, Sirintorn Chansirikarnjana, Pichai Ittasakul, Tipanetr Ngamkala, Lalita Sukumalin, Piangporn Charernwat, Krittika Saranburut, Taweevat Assavapokee

**Affiliations:** 1 Division of Geriatric Medicine, Department of Medicine, Faculty of Medicine, Ramathibodi Hospital, Mahidol University, Bangkok, 10400 Thailand; 2 Department of Psychiatry, Faculty of Medicine, Ramathibodi Hospital, Mahidol University, Bangkok, Thailand; 3 Department of Nursing, Faculty of Medicine, Ramathibodi Hospital, Mahidol University, Bangkok, Thailand; 4 Ramathibodi School of Nursing, Faculty of Medicine, Ramathibodi Hospital, Mahidol University, Bangkok, Thailand; 5 Cardiovascular and Metabolic Center, Faculty of Medicine, Ramathibodi Hospital, Mahidol University, Bangkok, Thailand

**Keywords:** Delirium, Prevention, Quetiapine, Older, Hospitalized

## Abstract

**Background:**

Delirium is a common disorder among hospitalized older patients and results in increased morbidity and mortality. The prevention of delirium is still challenging in older patient care. The role of antipsychotics in delirium prevention has been limited. Therefore, we conducted a trial to investigate the efficacy of quetiapine use to prevent delirium in hospitalized older medical patients.

**Methods:**

This study was a randomized double-blind controlled trial conducted at Ramathibodi Hospital, Bangkok, Thailand. Patients aged ≥65 years hospitalized in the internal medicine service were randomized to quetiapine 12.5 mg or placebo once daily at bedtime for a maximum 7-day duration. The primary end point was delirium incidence. Secondary end points were delirium duration, length of hospital stay, ICU admission, rehospitalization and mortality within 30 and 90 days.

**Results:**

A total of 122 patients were enrolled in the study. Eight (6.6%) left the trial before receiving the first dose of the intervention, whereas 114 (93.4%) were included in an intention-to-treat analysis allocated to the quetiapine or placebo group (*n* = 57 each). The delirium incidence rates in the quetiapine and placebo groups were 14.0 and 8.8% (OR = 1.698, 95% CI 0.520–5.545, *P* = 0.381), respectively. Other endpoints in the quetiapine and placebo groups were the median length of hospital stay, 6 (4–8) days versus 5 (4–8) days (*P* = 0.133), respectively; delirium duration, 4 (2.3–6.5) versus 3 (1.5–4.0) days (*P* = 0.557), respectively; ICU admission, 3 (5.3%) patients from both groups (*P* = 1.000); and mortality in the quetiapine and placebo groups, 1 (1.8%) versus 2 (3.5%) at 30 days (*P* = 0.566) and 7 (12.3%) versus 9 (15.8%) days at 90 days (*P* = 0.591). There were no significant differences in other outcomes. None of the participants reported adverse events.

**Conclusions:**

Quetiapine prophylaxis did not reduce delirium incidence in hospitalized older medical patients. The use of quetiapine to prevent delirium in this population group should not be recommended.

**Trial registration:**

This trial was retrospectively registered with the Thai clinical trials registry (TCTR) at clinicaltrials.in.th (TCTR20190927001) on September 26, 2019.

**Supplementary Information:**

The online version contains supplementary material available at 10.1186/s12877-021-02160-7.

## Background

Acute delirium is one of the most common clinical syndromes in hospitalized older patients. The features of delirium include acute onset and disturbances in attention and cognition [1]. A previous systematic review found the overall prevalence of delirium on admission to range between 10 and 31% and the overall rate to range between 11 and 42% during the hospital stay [2]. In geriatric wards, Inouye SK et al. found that the prevalence and incidence of delirium were 25% and 20–29%, respectively, which were much higher than those in general medical wards [3].

Older patients who developed delirium were associated with a 1.9x increased risk of mortality, a 1.3x increased risk of falls, and a 2.5x increased risk of institutionalization [3]. Furthermore, delirium was found to be related to an increased length of hospital stay. Some patients still had persistent symptoms of delirium at discharge and at 6 and 12 months. Mortality was also increased at 12 months after discharge [2].

The development of delirium is dependent on the interrelationship between a vulnerable patient with predisposing factors and exposure to precipitating factors. Some predisposing factors include age 65 years or older, cognitive impairment or dementia, history of delirium, functional impairment, visual or hearing impairment, comorbidity or severity of illness, depression, and history of stroke and alcohol abuse. Factors that can precipitate delirium include psychoactive or sedative drugs, use of physical restraints, urinary catheterization, infection and surgery [3, 4].

Delirium is preventable in 30–40% of cases [3, 5]. Recent meta-analyses have demonstrated that multicomponent nonpharmacological interventions were effective in reducing the incidence of delirium [6, 7]. The Hospital Elder Life Program (HELP) is an example of a successful intervention model [8, 9].

Pharmacological interventions have also shown some benefit. A study using ramelteon, a melatonin agonist, demonstrated the prevention of delirium in patients who were admitted due to serious medical problems [10]. Role of antipsychotics has also been studied. Although the recent meta-analyses have shown first-generation antipsychotics (e.g., haloperidol) could not prevent delirium, second-generation antipsychotics (e.g., olanzapine and risperidone) tended to reduce the incidence of delirium in postoperative patients. However, the routine use of antipsychotics to prevent delirium has not yet recommended because of insufficient evidence and potential adverse events [11, 12].

Quetiapine, which is one of second-generation and preferable antipsychotics in treating delirium in older patients due to reduced extrapyramidal side effects with equal efficacy [13–17], has limited data on delirium prevention. To our knowledge, there has been no study on quetiapine in hospitalized internal medicine patients.

Therefore, in our study, we aimed to evaluate the efficacy of quetiapine for the prevention of delirium in hospitalized older patients.

## Method

### Trial design

We conducted a single-center, double-blind, randomized, placebo-controlled study at Ramathibodi Hospital, Mahidol University, Bangkok, Thailand. Participants were recruited between August 2018 and December 2018. The study was approved by the certified Medical Ethics Committee of the Faculty of Medicine, Ramathibodi Hospital, Mahidol University. Generic quetiapine was supplied by the pharmaceutical department of Ramathibodi Hospital.

The hospital pharmacy prepackaged the study drug and placebo into identical capsules, carried out the randomization, and blinded the investigators and subjects. An independent data and safety monitoring committee evaluated all potentially serious adverse events. The study was conducted according to the Declaration of Helsinki and Good Clinical Practice guidelines. The trial was retrospectively registered with the Thai clinical trials registry (TCTR) at clinicaltrials.in.th (TCTR20190927001). The study adheres to CONSORT guidelines.

### Participants

Eligible participants were patients aged ≥65 years and acutely hospitalized in a medical specialty. All patients were recruited from the emergency medical observation unit and general medical wards. Patients provided written informed consent before participation. Exclusion criteria included patient or family refusal or patients who were already diagnosed with delirium, dementia, or severe Parkinson’s disease, patients who were critically ill, unable to take medication, unable to communicate, expected to be discharged within 24 h, needed emergency surgery, had terminal illness, were currently taking antipsychotics, or patients who had active cardiac conditions, history of epilepsy, substance dependence or abuse, a blood potassium level ≤ 3.0 mEq/L or a corrected QTc ≥ 500 msec from EKG.

### Randomization and interventions

Eligible and consenting patients were randomly assigned to quetiapine 12.5 mg tablets or placebo once daily at 9 pm for a maximum of seven consecutive days. The dosage of quetiapine was based on the recommended initial dose for delirium treatment in older patients [13]. Quetiapine was given once daily at bedtime due to sedating effects to aid sleep and cover the night time, when delirium usually developed [18–21]. The duration of the intervention was 7 days, which is the time during which most patients develop new-onset delirium [22]. Patients were randomized into the intervention or placebo group using fixed randomization schemes per site with a block size of 4 (1:1) according to a computer-generated randomization list.

Placebo and quetiapine were identical in appearance and packaged in identical medical envelopes containing 7 tablets with sequentially numbered labels, each with a unique study identification number. The medication was given by the nurse. Emergency unblinding was possible via 24-h contact with an on-call pharmacist, in case delirium developed or assigned intervention might affect patient care. Study staff, clinicians and participants were to remain blinded throughout the study.

### Outcomes

The primary outcome was delirium incidence within seven consecutive days after the intervention was initiated. Secondary outcomes were delirium duration, hospital length of stay, ICU admission, rehospitalization, and 30- and 90-day mortalities.

### Data collection

All patients were interviewed and assessed by trained clinicians and investigators. Baseline demographic and health-related characteristics were recorded. Delirium was assessed at baseline by experienced clinicians or investigators. Patients who were diagnosed with delirium at baseline or had medical records suspecting delirium during admission were excluded from the study. During the study intervention period, all patients were assessed daily by clinicians or investigators. Patients were also observed by nursing and medical staff. A session of delirium assessment training based on the confusion assessment method (CAM) [23] was provided for the nurses in every participating ward. A document consisting of a questionnaire based on CAM [24] was attached to the patient’s medical chart and filled in by the investigator or nurses to monitor and record the symptoms. Medical and nursing records were also reviewed for evidence of delirium symptoms. Delirium diagnosis was confirmed by a clinician or investigator according to the Diagnostic and Statistical Manual of Mental Disorders, 5th edition (DSM-V) criteria for delirium [25].

When delirium was diagnosed, the intervention was unblinded. The patient was further investigated for all potential causes and received standard delirium treatment. The patient remained under follow-up until discharge. Medical records from every patient were reviewed after discharge and at 3 months to collect data on primary diagnosis, comorbidities, complications, ICU admission, readmission and mortalities. Safety was monitored throughout the study by daily observation, examination by clinicians and investigators and by patient report. The study would be immediately terminated and unblinded in the case of suspected adverse events.

### Sample size

Sample size was calculated based on the assumptions that the incidence of delirium in the placebo group would be 30% and that quetiapine prophylaxis would result in an absolute risk reduction of 20%. The estimate of the incidence of delirium in the placebo group was based on a previous study in a geriatric ward, which reported incidence rates ranging from 20 to 29% [3]. To detect a significant difference between groups, we sought to randomize 118 patients into 2 groups of 59 patients per treatment arm to give 80% statistical power at a two-sided 5% significance level (alpha).

### Statistical analysis

Statistical analyses were performed using IBM SPSS Statistics version 23. Descriptive statistics [mean ± standard deviation (SD)], frequency and percentage, or median and interquartile range (IQR) were used to describe baseline patient characteristics. Intention-to-treat (ITT) analysis was performed. Patients who did not develop delirium during the 7-day intervention period or left the study in case of early termination or hospital discharge were regarded as negative on the primary outcome.

The incidence of delirium was based on the number of participants who developed at least one delirium episode within the first 7 days after study initiation. The incidence of delirium was compared between groups using the Chi-squared test. Odds ratios (ORs) with 95% confidence intervals (CIs) were reported as effect sizes using placebo as a reference group. Secondary outcomes were compared by using the chi-square test or Fisher’s exact test for dichotomous and nominal outcomes, the independent-samples *t*-test for normally distributed continuous outcomes, the Mann-Whitney *U*-test for ordinal outcomes and continuous outcomes that were not normally distributed and the Hodges-Lehmann estimator for confidence intervals for the difference between 2 medians. *P*-values < 0.05 were considered statistically significant. Survival analyses presented by Kaplan-Meier curves were used for graphical demonstration. Cox proportional hazard regression analyses were performed to estimate the hazard ratios (HRs) for 30- and 90-day survival for the quetiapine and placebo groups. Sensitivity analyses were performed with age and length of hospital stay groups. Age groups were stratified by age less than 75 years and 75 years and over according to the mean age (75.3 ± 7.1 years) from our study baseline characteristics. Length of hospital stay were stratified by less than or equal to 5 days and over 5 days groups according to median length of hospital (5 days) from our study outcomes. Interaction was tested by subgroup.

## Results

### Enrollment and baseline data

**Fig. 1:**
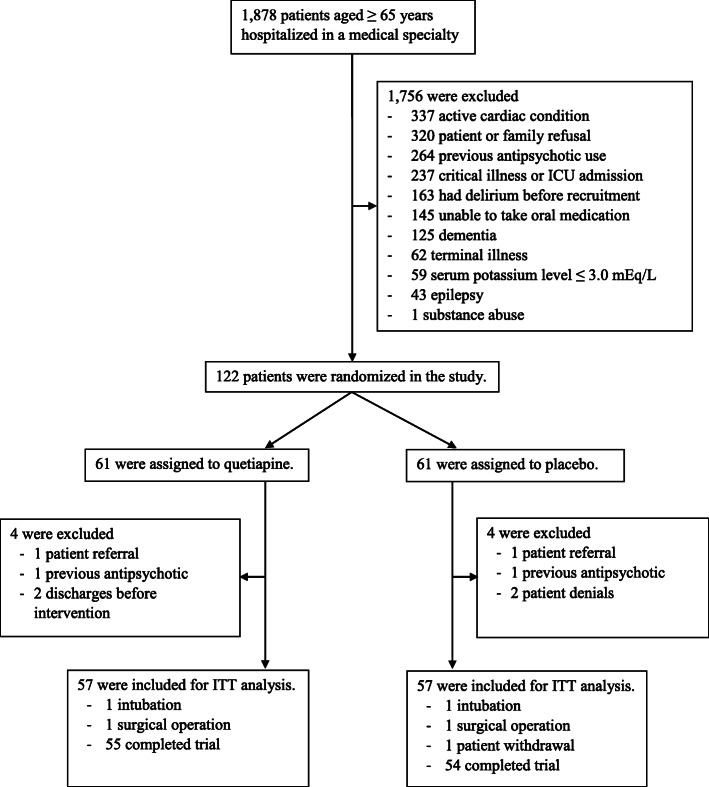
Study flow diagram

From August 2018 to December 2018, 1878 eligible patients aged over 65 years were admitted to a medical specialty. A total of 1756 patients were excluded, mostly due to active cardiac condition. A total of 122 participants were randomly assigned to quetiapine (*n* = 61) or placebo (n = 61). Eight participants (6.6%) were excluded (4 from each group) because of previous antipsychotic use, patient referral, patient or their relative’s denial and discharge before intervention initiation. A total of 114 participants (93.4%) (57 from each group) were included for ITT analysis of the primary outcome. However, 55 participants in the intervention group and 54 participants in the placebo group completed the trial. **(**Fig. 1**)** Three participants died during admission, and 4 participants died within the 3-month follow-up. The baseline characteristics of the quetiapine and placebo groups were not significantly different, as demonstrated in Table 1. Other baseline laboratory findings, previous comorbidities, and primary diagnoses of hospital admissions are described in the Supplementary Materials (Tables 1, 2 and 3 in Supplementary Materials), which were quite similar between both groups.

**Table 1:** Baseline characteristics of total study population (intention-to-treat)

	Placebo (***n*** = 57)	Quetiapine (***n*** = 57)	All (***n*** = 114)	***P***-Value
**Male**, n (%)	30 (52.6)	32 (56.1)	62 (54.4)	0.707
**Age**, mean (SD)	75.2 ± 6.9	75.4 ± 7.5	75.3 ± 7.1	0.835
< 75, n (%)	28 (49.1)	26 (45.6)	54 (47.4)	0.708
≥ 75, n (%)	29 (45.6)	31 (54.4)	60 (52.6)	
**Body weight (kg)**, mean (SD)	56.1 ± 10.4	55.7 ± 11.0	55.9 ± 10.6	0.851
**BMI (kg/m**^**2**^**)**, mean (SD)	22.56 ± 4.0	21.9 ± 3.8	22.2 ± 3.9	0.366
**CCI score**, median (IQR)	2 (1, 3)	2 (1, 3)	2 (1, 3)	0.584
**Prehospital living**, n (%)				0.079
Home	57 (100.0)	54 (94.7)	111 (97.4)	
Home care	0 (0.0)	3 (5.3)	3 (2.6)	
**Mobility status**, n (%)				0.050
Independent	42 (73.7)	32 (56.1)	74 (64.9)	
Gait aids	15 (26.3)	25 (43.9)	40 (35.1)	
**Hearing aids**, n (%)	3 (5.3)	3 (5.3)	6 (5.3)	1.000
**Visual aids**, n (%)	31 (54.4)	33 (57.9)	64 (56.1)	0.706
**Smoking**, n (%)	9 (15.8)	8 (14.0)	17 (14.9)	0.793
**Alcohol**, n (%)	3 (5.3)	4 (7.0)	7 (6.1)	0.927
**Previous medication**, n (%)				0.674
Anticholinergics	3 (5.3)	5 (8.8)	8 (7.0)	
Antihistamines	2 (3.5)	1 (1.8)	3 (2.6)	
Benzodiazepines	5 (8.8)	5 (8.8)	10 (8.8)	
Opioids	0 (0.0)	2 (3.5)	2 (1.8)	
**Blood test results**				
Albumin, g/dL, median (IQR)	29.9 (25.9, 34.5)	30.3 (25.0, 34.9)	30.1 (25.3,34.7)	0.629
Potassium, mmol/L, mean (SD)	4.2 ± 0.5	4.2 ± 0.9	4.2 ± 0.7	0.751
Creatinine, mg/dL, median (IQR)	0.87 (0.74, 1.40)	0.92 (0.68, 1.44)	0.91 (0.72, 1.41)	0.476
eGFR, ml/min/1.73m^2^, median (IQR)	73.1 (47.2, 88.6)	72.5 (45.5, 86.4)	72.6 (45.5, 88.2)	0.571
**Baseline QTc, ms**, mean (SD)	442.8 ± 30.5	446.2 ± 32.8	444.5 ± 31.6	0.562

n number, *P P*-value, *SD* Standard Deviation, *BMI* body mass index, kg/m^2^ kilograms per square meter, *CCI* Charlson comorbidity index, *QIR* Interquartile range, albumin 3.5–5.0 g/dL, potassium 3.50–5.10 mmol/L, creatinine 0.55–1.02 mg/dL

### Primary outcome

The incidence of delirium in the quetiapine group was 14.0%, while that in the placebo group was 8.8% (OR = 1.698, 95% CI 0.520–5.545, *P* = 0.381). (Table 2).

**Table 2:** Study outcome (intention-to-treat analysis)

Outcome	Placebo	Quetiapine	OR or Difference (95% CI)	***P***-value
Delirium incidence, n (%)	5 (8.8)	8 (14.0)	OR = 1.698 (0.520–5.545)	0.381
Length of hospital stay (day), median (IQR)	5 (4, 8)	6 (4, 8)	Difference = 0.0 (−1,0)*	0.133
Admission to participation day (day), median (IQR)	1 (1, 2)	1 (1, 2)	Difference = 0.0 (0,0)*	0.838
Admission to delirium day, median (IQR)	2 (1.0,4.5)	3.5 (2.0,4.0)	Difference = 0.0 (0,0)*	0.361
Delirium duration (day), median (IQR)	3 (1.5,4.0)	4 (2.3,6.5)	Difference = 0.0 (0,0)*	0.557
Total participation (day), median (IQR)	2 (1, 4)	3 (1, 4)	Difference = 0.0 (0,0)*	0.190
ICU admission, n (%)	3 (5.3)	3 (5.3)	OR = 1.000 (0.193–5.177)	1.000
Rehospitalization within 90 days after discharge	23 (40.4)	24 (42.1)	OR = 1.075 (0.510–2.267)	0.849
Mortality, n (%)				
At 30 days	2 (3.5)	1 (1.8)	OR = 0.491 (0.043–5.573)	0.566
At 90 days	9 (15.8)	7 (12.3)	OR = 0.747 (0.258–2.164)	0.591

*OR* Odds Ratios, *CI* Confidence Interval, *P P*-value, *QIR* Interquartile range, *ICU* Intensive Care Unit, *Differences between medians using Hodges-Lehmann estimator

### Secondary outcome

**Fig. 2:**
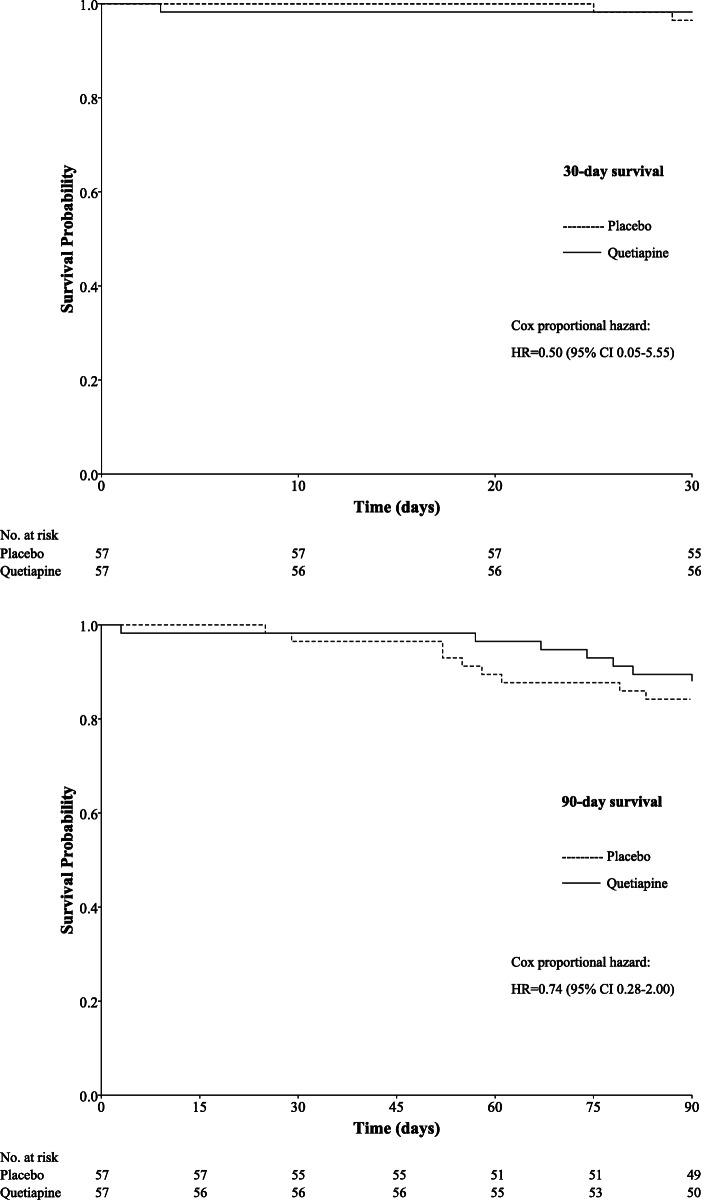
Survival analysis at 30 and 90 days

The median durations of delirium in the quetiapine and placebo groups were 4 (2.3–6.5) days and 3 (1.5–4.0) days, respectively, difference = 0 day (95% CI 0–0, *P* = 0.557). The median lengths of hospital stay were 6 (4-8) days and 5 (4-8) days in the intervention and placebo groups, respectively, difference = 0 day (95% CI 0–0, *P* = 0.133). Three patients (5.3%) from each group were transferred to the intensive care unit, OR = 1.000 (95% CI 0.193–5.177, *P* = 1.000) The rates of rehospitalization within 90 days after discharge were not significantly different between the two groups, which involved 24 (42.1%) and 23 (40.4%) patients in the intervention and placebo groups, OR = 1.075 (95% CI 0.510–2.267, *P* = 0.849), respectively. Mortality within 30 days affected 1 (1.8%) and 2 (3.5%) patients in the quetiapine and placebo groups, OR = 0.491 (95% CI 0.043–5.573, *P* = 0.566), respectively, whereas mortality within 90 days affected 7 (12.3%) and 9 (15.8%) patients in the quetiapine and placebo groups, OR = 0.747 (95% CI 0.258–2.164, *P* = 0.591), respectively. Survival analyses showed no difference between the quetiapine and placebo groups, HR = 0.50 (95% CI 0.05–5.55) and HR = 0.74 (95% CI 0.28–2.00) for 30- and 90-day survival, respectively. **(**Fig. 2**)** There were no reports of any adverse events during the current study period.

### Subgroup analysis

Sensitivity analyses were performed according to age group and length of hospital stay. No significant interaction between any of the subgroups and treatment was found. Delirium incidences and 30- and 90-day mortalities across both tested subgroups showed no significant differences between patients who received quetiapine and placebo. **(**Table 3**).**

**Table 3:** Sensitivity analysis

	Placebo (n = 57)	Quetiapine (***n*** = 57)	OR (95% CI)	Interaction Effect, ***P***-value
**Age group**				
**< 75 years (*n*** **= 54)**	*n* = 28	*n* = 26		.481 for delirium incidence 1.0 for 30-day mortality 0.057 for 90-day mortality
Delirium incidence, n (%)	3 (10.7)	3 (11.5)	1.09 (0.20–5.94)	
Mortality, n (%)				
30 days	2 (7.1)	1 (3.8)	0.52 (0.04–6.10)	
90 days	7 (25.0)	2 (7.7)	0.25 (0.05–1.34)	
**≥ 75 years (*n*** **= 60)**	*n* = 29	*n* = 31		
Delirium incidence, n (%)	2 (6.9)	5 (16.1)	2.60 (0.46–14.59)	
Mortality, n (%)				
30 days	0 (0.0)	0 (0.0)	0.00	
90 days	2 (6.9)	5 (16.1)	2.60 (0.46–14.59)	
**Length of hospital stay**				
**≤ 5 days (*n*** **= 61)**	*n* = 35	*n* = 26		.938 for delirium incidence .998 30-day mortality .088 for 90-day mortality
Delirium incidence, n (%)	2 (5.7)	2 (7.7)	1.38 (0.18–10.46)	
Mortality, n (%)				
30 days	1 (1.6)	0 (0.0)	0.00	
90 days	3 (8.6)	4 (15.4)	1.94 (0.40–9.53)	
**> 5 days (*n*** **= 53)**	*n* = 22	n = 31		
Delirium incidence, n (%)	3 (13.6)	6 (19.4)	1.52 (0.34–6.87)	
Mortality, n (%)				
30 days	1 (4.5)	1 (3.2)	0.70 (0.04–11.83)	
90 days	6 (27.3)	3 (9.7)	0.29 (0.06–1.30)	

*n* number, *OR* Odds ratios, *CI* Confidence Interval, *P P*-value

## Discussion

Antipsychotic prophylaxis for delirium prevention has been studied in a few trials. Most of the studies have focused on surgical and postoperative or critically ill patients. However, the results remain uncertain [11, 12, 14, 26–28]. The evidence for the role of antipsychotics in older medical patients has also been limited. A recent study using haloperidol versus placebo in acutely hospitalized older patients in general medicine or surgical specialties showed no differences in delirium incidence [29]. To our knowledge, this is the first study focusing on quetiapine use for delirium prevention in hospitalized older medical patients.

In our study, a randomized, double-blind, placebo-controlled trial, we found no positive outcome on delirium incidence in older medical patients admitted to the hospital. The secondary outcomes were nonsignificant between both groups. This result was similar to a previous study on haloperidol prophylaxis that included both medical and surgical patients [29].

Nonetheless, our study had some limitations. First, the delirium incidence rates in both groups were only 11.4%, which were much lower than the previous reference study, which had 20–29% incidence rates [3]. This might be because of our strict inclusion and exclusion criteria and hypoactive delirium underdetection. Those who were critically ill, had terminal illnesses, had active cardiac conditions, were unable to communicate or had dementia, which are high-risk groups for delirium, were not included in our study. Moreover, we did not find any hypoactive delirium incidence. It was possible that the event was underdetected because daily assessment and medical record review might not be sensitive enough to detect all occurrences. The majority of the participants were quite physically fit, and almost all patients could ambulate before admission, mostly without gait aids. In addition, due to certain unknown effects of antipsychotics (quetiapine in this study) on this particular population, we could not obtain accurate sample numbers. Therefore, we could not reach statistical power from our expected sample calculation.

Second, the dosage and administration of the intervention drug might influence the results. In this study, we administered only 12.5 mg of quetiapine once daily, which is the initial starting dosage for older patients. Those with strong significant precipitating factors such as sepsis or deoxygenation possibly needed a higher dosage of quetiapine to prevent delirium. Moreover, once daily administration at bedtime may not be an adequate treatment due to the short half-life of quetiapine, which is typically 6 h [30].

In addition, unlike surgical or critically ill patient groups who have significant precipitating factors for delirium, our population had various medical conditions with different disease severities, which could affect delirium incidences. We did not assess or collect data about the severity of diseases, although our report on primary diagnoses showed quite similar results between the two groups. Previous comorbidities were also collected and showed no difference at baseline. In subgroup analyses, patients with longer hospitalization might have worse medical conditions; however, the results remained nonsignificant.

Finally, we did not have data on cognition. We did not collect data on cognitive testing at baseline because of unreliable interpretation in acutely ill patients. Those with previously diagnosed dementia were excluded at the beginning. Nevertheless, cognition had a great impact on delirium outcome. Older patients with cognitive impairment or dementia had a higher rate of delirium during hospital admission [3], which could possibly affect our outcomes.

Although this study did not demonstrate a benefit for quetiapine prophylaxis in preventing delirium in older adults during hospital admission, the study provokes some further questions. Further trials focusing on different antipsychotics such as olanzapine, risperidone or other medications such as trazodone with various dosages and administration methods should be conducted. The study population might be narrowed down to one specified disease with similar comorbidities or stratified into disease categories and severities to see the potential effect of the medication within different patient groups. Moreover, delirium severity should also be collected as an additional outcome which could provide a sensitive continuous measurement of delirium between the groups.

## Conclusion

Our study demonstrated that quetiapine prophylaxis did not reduce the incidence of delirium in hospitalized older medical patients. The length of hospital stay, delirium duration, ICU admission, mortality and rehospitalization and mortality rates within 90 days after discharge were not significantly different between the intervention and control groups. In conclusion, quetiapine may not have benefit for preventing delirium in this population group.

## Supplementary Information

**Additional file 1: Table 1**. Baseline laboratory characteristics (Intention-to-treat). **Table 2**. Baseline comorbidities (intention-to-treat). **Table 3**. Primary diagnosis of participants (intention-to-treat). **Table 4**. Baseline characteristics of delirious participants.

## Data Availability

All datasets generated and analyzed during the current study are available in the figshare.com repository, 10.6084/m9.figshare.13182947

## Abbreviations

BMI: Body mass index
CAM: Confusion Assessment Method
CI: Confidence interval
EKG: Electrocardiography
HR: Hazard ratio
ICU: Intensive care unit
ITT: Intention-to-treat
kg/m2: Kilogram per square meter
mEq/L: Milliequivalents per liter
msec: Millisecond
N: Number
OR: Odds ratio
*P*: *P* value
QIR: Interquartile range
QTc: Q-T corrected (corrected Q-T interval)
SD: Standard deviation
