# Supplementary material for: Efficacy of quetiapine for delirium prevention in hospitalized older medical patients: a randomized double-blind controlled trial
Source: BMC Geriatr. 2021 Mar 31;21:215. doi: 10.1186/s12877-021-02160-7 (PMC8010962; doi:10.1186/s12877-021-02160-7)
Supplement: Supplementary file 1 — Additional file 1: Table 1. Baseline laboratory characteristics (Intention-to-treat). Table 2. Baseline comorbidities (intention-to-treat). Table 3. Primary diagnosis of participants (intention-to-treat). Table 4. Baseline characteristics of delirious participants. [file 12877_2021_2160_MOESM1_ESM.docx]

**Supplementary**

Table 1. Baseline laboratory characteristics (intention-to-treat)

Table 2. Baseline comorbidities (intention-to-treat)

Table 3. Primary diagnosis of participants (intention-to-treat)

Table 4. Baseline characteristics of delirious participants

|  | **Unit** | **Reference range** | **Placebo** | **(n)** | **Quetiapine** | **(n)** | **All** | **(n)** | ***P*-value** |
| --- | --- | --- | --- | --- | --- | --- | --- | --- | --- |
| White Blood Cells (WBC) | 10^3/uL | 4.00 - 10.00 | 8.8 (6.0, 13.6) | 57 | 8.4 (5.8, 13.5) | 57 | 8.6 (6.0, 13.4) | 114 | 0.72 |
| Hemoglobin (Hb) | g/dL | 12.00 - 16.00 | 11.2 (9.4, 13.15) | 57 | 11.7 (10.1, 12.8) | 57 | 11.5 (9.7, 12.9) | 114 | 0.65 |
| Hematocrits (Hct) | % | 36.00 - 48.00 | 33.19 ± 7.82 | 57 | 34.2 ± 7.01 | 57 | 33.76 ± 7.41 | 114 | 0.42 |
| Platelets (Plt) | 10^3/uL | 140.0 - 450.0 | 202.0 (139.5, 323.5) | 57 | 200.0 (144.0, 258.5) | 57 | 201.0 (144.3, 289.0) | 114 | 0.57 |
| Blood Urea Nitrogen (BUN) | mg/dL | 7 - 18 | 17.0 (12.0, 25.5) | 57 | 17.0 (13.5, 33.0) | 57 | 17.0 (13.0, 30.5) | 114 | 0.47 |
| Sodium (Na) | mmol/L | 136 - 145 | 137.0 (132.0, 139.0) | 57 | 136.0 (132.0, 139.0) | 57 | 136.0 (132.0, 139.0) | 114 | 0.87 |
| Chloride (Cl) | mmol/L | 98 - 107 | 103.0 (98.0, 106.5) | 57 | 102.0 (98.0, 105.5) | 57 | 102.5 (98.0, 106.0) | 114 | 0.32 |
| Carbon Dioxide (CO2) | mmol/L | 22.0 - 29.0 | 21.0 (18.5, 23.7) | 57 | 21.5 (19.1, 24.0) | 57 | 21.1 (18.8, 23.9) | 114 | 0.27 |
| Corrected Calcium (Ca) | mg/dL | 8.4 - 10.2 | 9.5 (9.0, 9.9) | 37 | 9.7 (9.3, 9.9) | 27 | 9.6 (9.1, 9.9) | 64 | 0.69 |
| Magnesium (Mg) | mg/dL | 1.6 - 2.6 | 2.1 (1.8, 2.3) | 35 | 2.0 (1.6, 2.2) | 31 | 2.0 (1.8, 2.2) | 66 | 0.16 |
| Phosphate (PO4) | mg/dL | 2.3 - 4.7 | 3.37 ± 0.66 | 35 | 3.21 ± 1.28 | 27 | 3.30 ± 0.97 | 62 | 0.53 |
| Total bilirubin (TB) | mg/dL | 0.2 - 1.2 | 0.8 (0.5, 1.2) | 42 | 0.7 (0.5, 1.0) | 50 | 0.8 (0.5, 1.2) | 92 | 0.57 |
| Direct bilirubin (DB) | mg/dL | 0.0 - 0.5 | 0.3 (0.2, 0.5) | 42 | 0.3 (0.2, 0.5) | 50 | 0.3 (0.2, 0.5) | 92 | 0.58 |
| Aspartate Aminotransferase (AST) | U/L | 5 - 34 | 42.0 (30.0, 72.0) | 43 | 20.0 (28.0, 66.5) | 50 | 41.0 (29.5, 67.0) | 93 | 0.50 |
| Alanine Transaminase (ALT) | U/L | 0 - 55 | 27.0 (16.0, 53.0) | 43 | 27.5 (13.0, 42.3) | 50 | 27.0 (13.0, 47.0) | 93 | 0.85 |
| Alkaline Phosphatase (ALP) | U/L | 40 - 150 | 93.0 (76.8, 113.3) | 42 | 94.0 (69.0, 171.3) | 50 | 93.0 (74.0, 134.5) | 92 | 0.25 |
| Total protein (TP) | g/dL | 6.4 - 8.3 | 6.69 ± 0.84 | 42 | 6.70 ± 1.37 | 50 | 6.70 ± 1.15 | 92 | 0.97 |
| Blood sugar (BS) | mg/dL | 70 - 100 | 117.0 (101.5, 142.5) | 53 | 121.0 (102.5, 145.3) | 52 | 118.0 (102.0, 143.0) | 105 | 0.76 |

**Table 1. Baseline laboratory characteristics (Intention-to-treat)**

Values expressed as mean ± SD or median (IQR); n number; *P* *P*-value

**Table 2. Baseline comorbidities (intention-to-treat)**

| **Comorbidity** | **Placebo (n=57)** | **Quetiapine (n=57)** | **All (n=114)** | ***P*-Value** |
| --- | --- | --- | --- | --- |
| Hypertension | 45 (78.9) | 44 (77.2) | 89 (78.1) | 0.821 |
| Diabetes | 19 (33.3) | 20 (35.1) | 39 (34.2) | 0.843 |
| Dyslipidemia | 35 (61.4) | 31 (54.4) | 66 (57.9) | 0.448 |
| Chronic kidney disease | 17 (29.8) | 24 (42.1) | 41 (36.0) | 0.172 |
| Old myocardial infarction | 10 (17.5) | 7 (12.3) | 17 (14.9) | 0.430 |
| Old cerebrovascular accident | 6 (10.5) | 7 (12.3) | 13 (11.4) | 0.768 |
| Asthma | 6 (10.5) | 5 (8.8) | 11 (9.6) | 0.751 |
| Chronic Obstructive Pulmonary Disease | 12 (21.1) | 9 (15.8) | 21 (18.4) | 0.469 |
| Old pulmonary tuberculosis | 11 (19.3) | 3 (5.3) | 14 (12.3) | 0.022 |
| Obstructive sleep apnea | 3 (5.3) | 1 (1.8) | 4 (3.5) | 0.309 |
| Chronic HBV infection | 3 (5.3) | 3 (5.3) | 6 (5.3) | 1.000 |
| Fatty liver | 1 (1.8) | 0 (0.0) | 1 (0.9) | 0.315 |
| Chronic cirrhosis | 3 (5.3) | 2 (3.5) | 5 (4.4) | 0.647 |
| Constipation | 3 (5.3) | 4 (7.0) | 7 (6.1) | 0.696 |
| Benign prostatic hyperplasia | 8 (14.0) | 7 (12.3) | 15 (13.2) | 0.782 |
| Osteoporosis | 3 (5.3) | 7 (12.3) | 10 (8.8) | 0.185 |
| Osteoarthritis of the knee | 8 (14.0) | 8 (14.0) | 16 (14.0) | 1.000 |
| Gouty arthritis | 7 (12.3) | 9 (15.8) | 16 (14.0) | 0.590 |
| Anemia | 12 (21.1) | 12 (21.1) | 24 (21.1) | 1.000 |
| Hypothyroidism | 2 (3.5) | 3 (5.3) | 5 (4.4) | 0.647 |
| Hyperthyroidism | 1 (1.8) | 0 (0.0) | 1 (0.9) | 0.315 |
| Syndrome of inappropriate antidiuretic hormone secretion | 2 (3.5) | 2 (3.5) | 4 (3.5) | 1.000 |
| Vitamin D deficiency | 4 (7.0) | 9 (15.8) | 13 (11.4) | 0.141 |
| Depression | 0 (0.0) | 1 (1.8) | 1 (0.9) | 0.315 |
| Cancers | 22 (38.6) | 16 (28.1) | 38 (33.3) | 0.233 |

Values expressed as number (%); n number; *P* *P*-value

**Table 3. Primary diagnosis of participants (intention-to-treat)**

| **Primary diagnosis** | **Placebo (n=57)** | **Quetiapine (n=57)** | **All (n=114)** |
| --- | --- | --- | --- |
| Respiratory tract infection | 27 (47.4) | 23 (40.4) | 50 (43.9) |
| Urinary tract infection | 2 (3.5) | 6 (10.5) | 8 (7.0) |
| Sepsis | 4 (7.0) | 4 (7.0) | 8 (7.0) |
| Hepatobiliary tract infection | 2 (3.5) | 5 (8.8) | 7 (6.1) |
| Hematologic disease | 3 (5.3) | 2 (3.5) | 5 (4.4) |
| Skin and soft tissue infection | 3 (5.3) | 2 (3.5) | 5 (4.4) |
| Stroke | 3 (5.3) | 2 (3.5) | 5 (4.4) |
| Gastrointestinal tract infection | 2 (3.5) | 1 (1.8) | 3 (2.6) |
| Acute febrile illness | 1 (1.8) | 1 (1.8) | 2 (1.8) |
| GI bleeding | 1 (1.8) | 1 (1.8) | 2 (1.8) |
| Pleural effusion | 0 (0.0) | 2 (3.5) | 2 (1.8) |
| Unspecified viral infection | 0 (0.0) | 2 (3.5) | 2 (1.8) |
| Septic arthritis | 1 (1.8) | 0 (0.0) | 1 (0.9) |
| Others | 8 (14.0) | 6 (10.5) | 14 (12.3) |
| **Total** | 57 (100.0) | 57 (100.0) | 114 (100.0) |

Values expressed as number (%); n number

**Table 4. Baseline characteristics of delirious participants**

|  | **Placebo (n=5)** | **Quetiapine (n=8)** | **All (n=13)** | ***P*-Value** |
| --- | --- | --- | --- | --- |
| **Male**, n (%) | 2 (40.0) | 4 (50.0) | 6 (46.2) | 0.725 |
| **Age**, mean (SD) | 76.6 ± 10.6 | 79.3 ± 7.0 | 78.2 ± 8.2 | 0.595 |
| <75, n (%) | 3 (60.0) | 3 (37.5) | 6 (46.2) | 0.429 |
| ≥75, n (%) | 2 (40.0) | 5 (62.5) | 7 (53.8) |  |
| **Body weight (kg)**, mean (SD) | 54.5 ± 12.7 | 54.8 ± 12.4 | 55.9 ± 10.6 | 0.968 |
| **BMI (kg/m^2^)**, mean (SD) | 22.56 ± 4.0 | 20.9 ± 3.4 | 21.3 ± 3.6 | 0.586 |
| **CCI score**, median (IQR) | 2 (0.5,3) | 2 (1,5.3) | 2 (1,3) | 0.443 |
| **Prehospital living**, n (%) |  |  |  | N/A |
| Home | 5 (100.0) | 8 (100.0) | 13 (100.0) |  |
| Home care | 0 (0.0) | 0 (0.0) | 0 (0.0) |  |
| **Mobility status**, n (%) |  |  |  | 0.053 |
| Independent | 4 (80.0) | 2 (25.0) | 6 (46.2) |  |
| Gait aids | 1 (20.0) | 6 (75.0) | 7 (53.8) |  |
| **Hearing aids**, n (%) | 0 (0.0) | 1 (12.5) | 1 (7.7) | 0.411 |
| **Visual aids**, n (%) | 1 (20.0) | 4 (50.0) | 5 (38.5) | 0.279 |
| **Smoking**, n (%) | 5 (100.0) | 6 (75.0) | 11 (84.6) | 0.224 |
| **Alcohol**, n (%) | 0 (0.0) | 0 (0.0) | 0 (0.0) | N/A |
| **Previous medication**, n (%) |  |  |  |  |
| Anticholinergics | 0 (0.0) | 2 (25.0) | 2 (15.4) | 0.224 |
| Antihistamines | 0 (0.0) | 0 (0.0) | 0 (0.0) | N/A |
| Benzodiazepines | 1 (20.0) | 3 (37.5) | 4 (30.8) | 0.506 |
| Opioids | 0 (0.0) | 2 (25.0) | 2 (15.4) | 0.224 |
| **Blood test results** |  |  |  |  |
| Albumin, g/dL, median (IQR) | 30.0 ± 4.4 | 24.9 ±10.3 | 26.7 ± 8.7 | 0.379 |
| Potassium, mmol/L, mean (SD) | 4.4 (3.8,4.5) | 4.2 (3.6,4.5) | 4.2 (3.8,4.5) | 0.675 |
| Creatinine**,** mg/dL, median (IQR) | 0.92 (0.70,1.76) | 0.66 (0.52,93) | 0.82 (0.60,1.15) | 0.129 |
| eGFR, ml/min/1.73m^2^, median (IQR) | 61.1 ± 24.1 | 81.9 ± 17.2 | 73.9 ± 21.9 | 0.095 |
| **Baseline QTc,** ms, mean (SD) | 462.2 ± 41.1 | 435.8 ± 20.6 | 445.9 ± 31.5 | 0.147 |

n number; *P* *P*-value; SD Standard Deviation; BMI body mass index; kg/m^2^ kilograms per square meter; CCI Charlson comorbidity index; QIR Interquartile range; albumin 3.5 – 5.0 g/dL; potassium 3.50 – 5.10 mmol/L; creatinine; 0.55 – 1.02 mg/dL
